# Microarray Scanner Performance Over a Five-Week Period as Measured With Cy5 and Cy3 Serial Dilution Slides

**DOI:** 10.6028/jres.113.012

**Published:** 2008-06-01

**Authors:** Mary B. Satterfield, Katrice Lippa, Z. Q. Lu, Marc L. Salit

**Affiliations:** National Institute of Standards and Technology, Gaithersburg, MD 20899

**Keywords:** cyanine dyes, instrument qualification, microarray, microarray scanner, validation

## Abstract

To investigate scanner performance and guide development of an instrument qualification method, slides with replicates of successive dilutions of cyanine 5 (Cy5) and cyanine 3 (Cy3) dyes (referred to as dye slides) were scanned. The successive dilutions form a dose-response curve from which performance can be assessed. The effects of a variety of factors, including the number of scans and slide storage conditions, on scanner performance over a five-week period were investigated and tracked with time series charts of dye signal intensity, signal-to-noise (S/N), signal background, slope, and limit of detection (LOD). Scanner drift was tracked with a known stable reference material, Standard Reference Material (SRM) 2242. The greatest effect on the figures of merit was the dye, with the Cy5 dye showing signs of degradation after one week of scanning independent of all other factors while the Cy3 dye remained relatively stable. Use of the charts to track scanner performance over time holds promise for development of a method for microarray scanner performance qualification. Although not a prescription for performance qualification, this introductory study provides sufficient information regarding the use of dye slides to enable the user to institute a preliminary test method.

## 1. Introduction

The advent of the microarray has made possible the study of complex mixtures of RNA, DNA, proteins, and other biological samples. These samples are tested against probes that often number in the tens of thousands for a single experiment. While microarrays make possible measurement of numerous different fluorescent-labeled targets all at one time, the measurement needs of the technology, including validation, uncertainty, and traceability, are still in the development stage [1]. Issues including reproducibility are under investigation in an effort to accelerate the maturation of the field and enable regulated applications [2]. Other groups are working to provide information regarding the technical performance of a microarray experiment through the use of spike-in materials [3].

The instrument used to measure the fluorescent signal of the hybridized sample, the microarray scanner, should not be overlooked as a source of variability in microarray experiments. The scanner is usually assumed to work in a consistent and predictable manner over a wide range of operating settings. Work investigating the influence of the microarray scanner on experimental results includes a study on the effective photomultiplier tube settings for optimal two-color ratios [4] as well as a related study in which a channel-specific bias was identified when the signal intensities were very low [5]. In both studies algorithms were developed for correction and use of an extended dynamic range. An additional study has confirmed the nonlinearity of dose-response curves at high and low signal intensities over a range of instrument gain settings [6].

Ideally the microarray scanner is expected to produce a consistent response to a stable material day after day, a response that matches that measured on the previous day and with the previous scan. However, without instrument qualification materials and a quantitative history of stable performance it is difficult to tell that a microarray scanner is working correctly. Scanner performance qualification would provide an on-going record of scanner performance allowing the user to estimate the variability the scanner is contributing to the microarray experiment. Although some studies have been carried out with a focus on optimization of scanner use [7–10], there are no methods reported regarding the qualification of microarray scanner performance.

The United States Food and Drug Administration (USFDA) has in place regulations for validation of analytical methods [11]. Validation of instrument performance, more correctly referred to as instrument qualification, is under development by many sectors, as the recognition of the importance of instrument performance in data confidence increases [12–14]. Suggested guidelines were recently published by the United States Pharmacopeia (USP) in a chapter, USP-NF <1058>, on analytical instrument qualification [15]. These guidelines list analytical instrument qualification as a fundamental component of data quality, underpinning analytical method validation, system suitability tests, and quality control check samples. Our team at the National Institute of Standards and Technology (NIST) is developing a method for microarray scanner performance qualification, designed to validate signal measurement in microarray experiments by providing quantitative, objective evidence that a scanner is performing consistently from day to day.

To qualify microarray scanner performance a material that produces the same response under given conditions over a defined time period is required. This permits assessment of performance separately from the performance of the material being scanned. In addition to stability, an ideal performance qualification tool should mimic a microarray with similar spot sizes, resolution, concentrations, and background signal intensity. The fluorescent material used on the tool should be well-characterized, with traceability to a standard. It should produce a signal in the range of that measured at the typical experimental wavelengths and operating conditions, e.g. photomultiplier gain and laser power. The material used should be available in differing con centrations, thus producing signals in a range of intensities. Production of the material in different concentrations would enable measurement of a calibration curve of changing signal intensities, with measurements of slope and LOD possible. The ideal instrument qualification material would be available in a wide range of concentrations to allow for measurements at different instrument settings and on different scanners. With a wide range of concentrations available, a region below the LOD could be measured and on-spot background determined. Additionally, a region in which the scanner is saturated, or has reached its upper signal limit, would be visible, thus making possible measurement of the dynamic range. The ideal material would be spread out in differing concentrations over the area typically used by a microarray in order to assess the ability of the scanner to detect signal differences between replicate spots over the entire scan range and scan area.

**Fig. 1:**
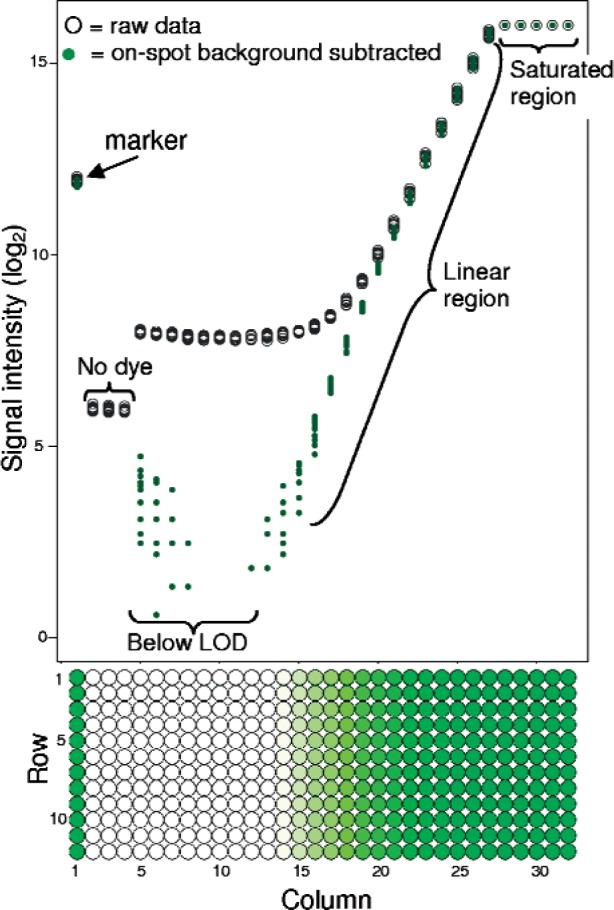
Layout of the cyanine dye slide (Cy3 block shown) and corresponding dose-response curve with raw and on-spot background subtracted data shown. The background subtracted value was measured as the median signal intensity of columns 5–10. Note the extended linear region possible with the background subtracted data.

Most microarray experiments employ the organic dyes, Cy5 and Cy3, or dyes with similar fluorescence spectra, for sample labeling, and most microarray scanners are configured for these dyes. The use of an array of serial dilutions of the Cy5 and Cy3 dyes with many of the characteristics of an ideal material was explored to measure scanner performance over a 5-week period. (See Fig. 1 for the dye layout of a single block.) Arrays of serial two-fold dilutions of the Cy5 and Cy3 dyes have been used to investigate the linearity of microarray scanners [16], and the characteristics of dose-response curves [6]. The inherent characteristics of these two dyes, including their photoinstability, do not permit traceability to a standard fluorescent material. (Serial dilution curves calculated using these dyes are therefore more properly referred to as dose-response curves and not calibration curves since the concentrations of the dyes cannot be related to a standard [17].)

**Fig. 2:**
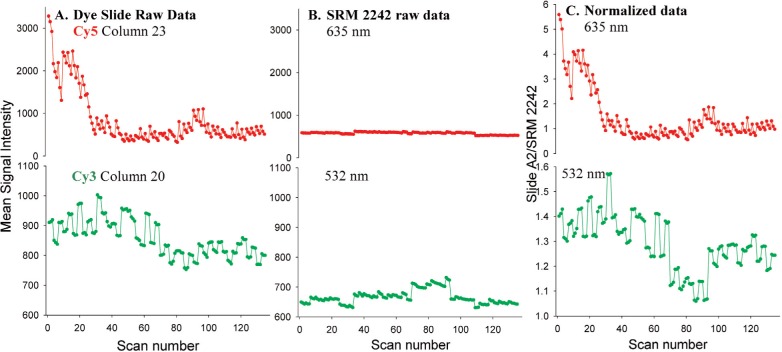
Effect of normalization of signal intensities of slide A2 with SRM 2242. **(A)** Raw data of a single column of slide A2. **(B)** Raw data of SRM 2242 used for comparison to dye slide data. **(C)** Normalized data: raw data of slide A2 divided by signal intensities of SRM 2242.

To assess the stability of the cyanine dyes relative to the instrument stability, a homogeneous fluorescent material, SRM 2242, was incorporated into the study. SRM 2242, a manganese oxide glass is certified for use at 488 nm, 514 nm, and 532 nm excitation wavelengths for Raman spectral correction and is typically used at much greater laser powers than used in a microarray scanner [18,19]. The SRM signal intensities were compared to those of a column of the dye of similar intensities. Panels A and B of Fig. 2 contain graphs of the signal intensity as a function of scan number over the five weeks of the study for a single column of each of the dye blocks and for SRM 2242. Panel C is a graph of this data after division of the dye slide intensities by the signal intensity of SRM 2242. This normalization process would remove any instrumental drift that appeared in both scans. The consistent gross shape of the right and left panels indicates that the dominant source of variability was in the fluorescence signal from the dye slides, and that the scanner was relatively stable throughout the study.

Scanner performance was evaluated over time with the use of the dye slides. Multiple scans were taken and the figures of merit that were candidates for scanner qualification were tracked. Figures of merit are performance characteristics used to evaluate the quality of a measurement or, as in this case, to qualify instrument performance. An ideal figure of merit would track instrument performance closely, and could be used to establish that the performance is in control against previously determined limits based on reference measurements. Useful figures of merit can often be related to some aspect of instrument performance or environmental aberration, and can be used as a diagnostic. The more stable a figure of merit, the smaller change in the instrument could be detected, making stable figures desirable. The figures of merit explored for the two dyes include signal intensity at a single concentration, S/N at a single concentration, on-spot background, slope of the dose-response curve (sensitivity), and LOD (see Table 1). Factors that might have an effect on the performance of the dye slides over time were investigated, including the effect of the number of scans performed, the recovery time allowed between scans, the elapsed time between slide production and use, and the storage of the slides in room air versus desiccation (see Table 2). Additionally, two different scanner models, referred to as Scanner R and Scanner S, were used in the study therefore making possible scanner-to-scanner assessment.

## 2. Methods and Materials

### 2.1 Experimental Plan

Slides in each lot were scanned at different intervals and stored under different conditions. Slides were scanned in triplicate, with each slide being scanned three times in a row before changing to another slide. To investigate the effect of the numbers of scans on the figures of merit, slides were divided into three categories of use (see Table 2). Heavy use slides, slides A2, B2, B3, and C2, were scanned every day in triplicate on Scanner R four days a week, morning and afternoon for a total of six scans/day. On the fifth day of the week the heavy use and standard use slides, slides A1, A3, B1, C1, and C3, were scanned in triplicate morning and afternoon, in the morning on one scanner and in the afternoon on the other scanner. The minimal use slides, slides A4, B4, and C4, were scanned in triplicate morning and afternoon on the first day of the study and again four weeks later in triplicate morning and afternoon on both scanner R and S, in the morning on one scanner and in the afternoon on the other scanner. To allow recovery of the dye after scanning, a five-minute lag time was allowed after each scan.

SRM 2242 was scanned three times in quick succession at the beginning of the morning and end of the afternoon. The SRM signal was measured by applying an analysis block of 12 columns and 32 rows, identical to that used with the dye slides. A single column of 12 spots on the SRM was chosen for comparison to the slide A2 signal. A column from each block of slide A2 was chosen that most closely matched the signal intensities of the SRM: column 23 in the Cy5 block and column 20 in the Cy3 block. For normalization, the mean intensity of a single column of slide A2 was divided by the mean signal intensity of a single column of the SRM (see Fig. 2).

To determine the effect of humidity on the dyes and on the figures of merit, most of the slides were stored in a desiccator per manufacturer specification. Slides A3, B3, B4, and C3 were not stored under desiccation. All slides were stored in their original containers in the dark at room temperature when not in use.

Slides were scanned in random order (as determined by a random number generator) and in alternating mornings and afternoons on days when both instruments were used.

Calibration, intended to maintain the signal intensity over time by adjusting the gain to the photomultiplier tube, was carried out with manufacturer-provided calibration slides on Scanner R, but not on Scanner S, due to the instrument being used for other purposes during this time. Scanner R was calibrated on the day before the 5-week study began. At the beginning of the study calibration constants were measured as 0.9781 at 635 nm and 1.1497 at 532 nm. As recommended by the manufacturer, Scanner R was calibrated again on Day 1 of Week 5 of the study. Calibration constants at that time were measured as 0.9622 at 635 nm and 1.1458 at 532 nm.

### 2.2 Data Analysis and Calculation of the Figures of Merit

The raw data in the form of tif files (available upon request) were converted to gpr files and then imported into R, a statistical computing software (http://www.r-project.org/). An R program was used to convert the data from the gpr files into a format conducive for plotting the figures of merit. The data were background subtracted after selecting the on-spot background as the median signal intensity of columns five through ten, all of which were below the LOD for the entirety of the study. After background subtraction the data was log_2_ transformed and a line fit to the data in the linear range by selecting only the columns that fit the following heuristic: the first acceptable column was one in which none of the 12 features exhibited a negative signal, and the last acceptable column was the one closest to the saturated columns but that did not have any saturated features. The fit was inverse-variance weighted, with columns with the smallest variance being accorded the greatest weight.

The LOD was calculated as the column at which the S/N = 3, with S/N being defined as the (signal – on-spot background)/standard deviation of the background. To convert from the signal intensity at the LOD to the column, the equation for the fit was used with the fit slope and y-intercept. Signal intensity of column 25 was measured as the mean of the 12 spots of the log_2_ intensities. To make sure that column 25 was representative of columns within the linear region several columns of data within the linear dose-response curve range were examined, and all showed similar signal variability. Column 25 was chosen for monitoring based on its position in the middle of the linear region. The signal intensity of column 25 was not background subtracted. The S/N used is that of the dye at column 25, but using a non-log_2_ transformed signal intensity and noise as the standard deviation of the mean of the 12 rows of the column. Percent change values were calculated from median values of the triplicates, using data that had not been log transformed.

To compare the effects of the factors used in this study and to compare slide to slide differences, analysis of variance (ANOVA) was used (S-Plus, Insightful Corporation) with a p-value of 0.05. To visually compare the figures of merit from all the scans a parallel coordinate plot was constructed using R.

### 2.3 Materials

Slides from Full Moon Biosystems (Sunnyvale, CA)^1^ with separate blocks of successive dilutions of Cy3 and Cy5 dyes were used including four slides from each of three different lots, with warranty expiration dates of September 2005 (lot C) and July 2006 (lots A and B). According to the manufacturer, the warranty expiration date of three months after printing is to account for replacement of slides with physical or performance defects. The slides are expected to produce stable signals for several months after the warranty expiration date. Manufacturing changes were made between the production of lot C and lots A and B in which the dye blocks were placed closer to the center of the slide and the distance between the spots decreased from 350 mm to 280 mm. No changes were made in the surface chemistry or dyes.

The five-week experiment was carried out in March and April of 2006 at which time lot C was past its warranty expiration date and lots A and B were well within the warranty period. The slides were scanned on two different models of microarray scanners from the same manufacturer, referred to as Scanner R and Scanner S. Scanner R was used daily (five days/week) and Scanner S was used one day/week. As suggested by the manufacturer of the dye slides, the laser power and gain of the scanner were optimized on the first dye slide scanned (one from lot C and one from lots A or B) on the first day of the study so that between four to six columns of the dye slides had saturated intensities. Although initial optimization efforts were made to select operating parameters that would produce similar signals across all slides, the signal on the lot C slides was slightly different than on the lots A and B slides. Settings used for the two scanners are shown in Table 3 and were held constant throughout the five-week study.

A single piece of SRM 2242 glass (NIST, Gaithersburg, MD), a manganese-doped (0.15 wt. % MnO_2_) borate matrix glass, was used throughout the study [18]. The SRM is 10.7 mm × 30.4 mm × 2.0 mm, and a holder the size of a microscope slide, 25 mm × 75 mm × 1 mm, was made to hold the SRM in place in the scanner. The holder was constructed from a polymethylmethacrylate (PMMA) sheet with a rectangle the size of the SRM glass cut out of the middle, into which the SRM was press-fit in place. Scans of the SRM were made on the smooth side of the glass; the frosted side of the glass is certified for Raman spectral correction. The smooth side of the glass was chosen for scanner measurements due to the decreased noise relative to the frosted side.

## 3. Results and Discussion

### 3.1 Experimental Design Overview

To track scanner performance over time, we used figures of merit derived or used directly from the dye slide image analysis data. One goal of this study was to observe the behavior of these figures of merit over time and determine which hold the greatest potential for development of specifications and quality attributes that indicate the microarray scanner is in control. As seen in Fig. 1, a dose-response curve was calculated for each scan as a composite of the 12 replicates of dye concentrations, and the slope and LOD calculated. Each column of spots corresponds to a single dye concentration, and as has been observed previously the curve is nonlinear at both the top and bottom sections [6]. As shown in Fig. 1, at most instrument settings the maximum signal is determined by the instrument saturation and occurs as an abrupt cutoff while the change in slope of the raw data near the bottom of the linear region appears more gradual as the slope changes from approximately 1 to 0. When background subtraction is applied prior to log_2_ transformation and calculation of the slope and LOD the dose-response curve changes at the LOD with an abrupt shift from signal to noise. Background subtraction results in an increased linear range and lowered LOD.

**Fig. 3:**
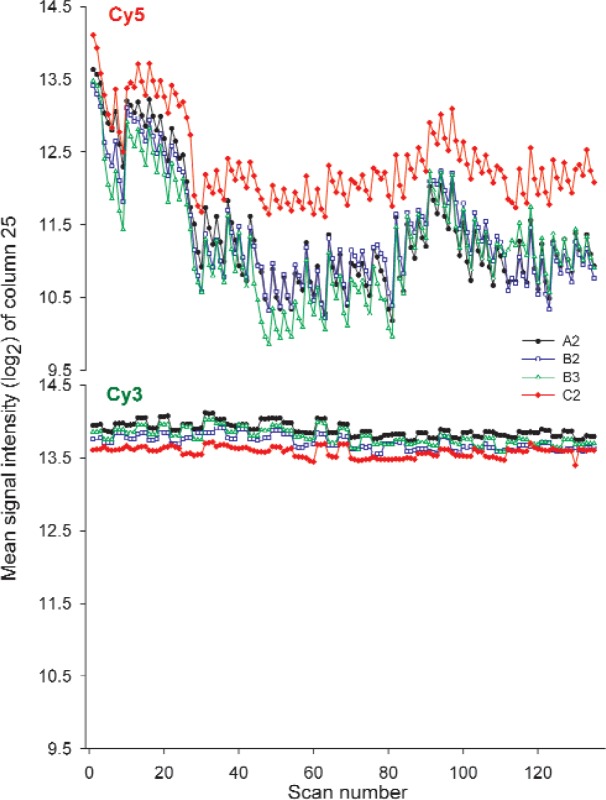
Graphs of column 25 signal intensity of heavy use slides over five weeks taken on Scanner R. Each point represents the median signal intensity of column 25 of a single scan. On four days of each week three successive scans were made mornings and afternoons on the same scanner with a five-minute lag between scans. On the fifth day three scans were made in the morning or afternoon on one scanner with the other three scans taken on the alternate scanner. The scans plotted represent 27 scans/week for five weeks.

**Fig. 4:**
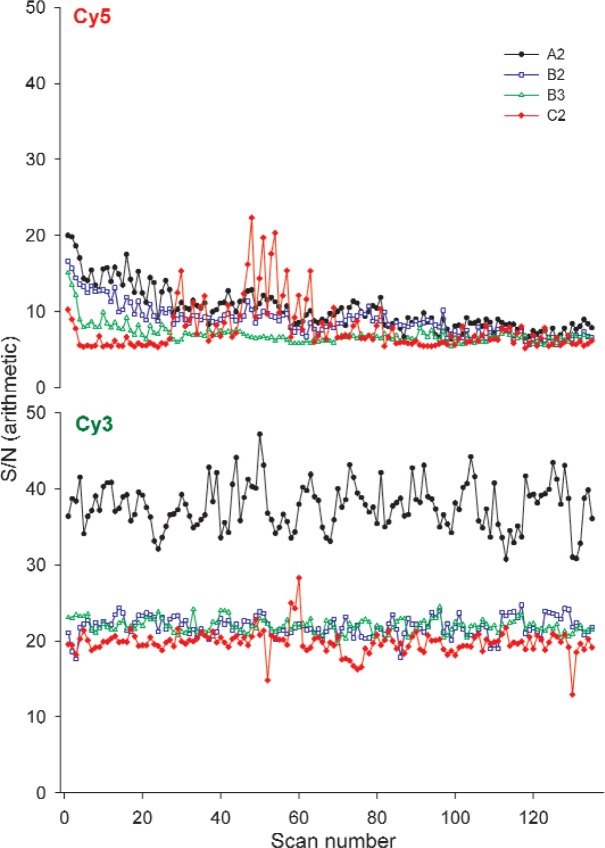
Graphs of the S/N of column 25 of heavy use slides over five weeks on Scanner R. Scans were taken in triplicate with a five-minute lag. The scans plotted represent 27 scans/week for five weeks.

**Fig. 5:**
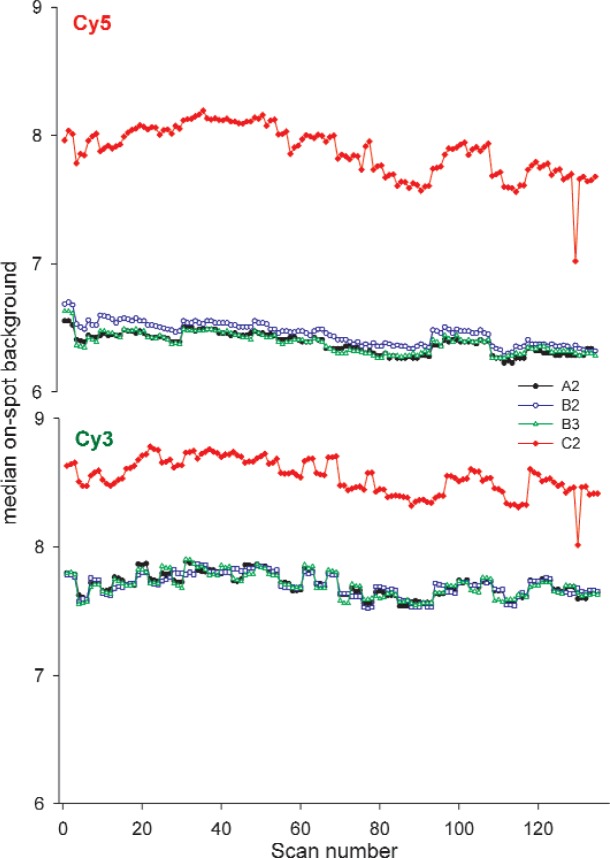
Graphs of the median on-spot background signal of heavy use slides over the five weeks of the study taken on Scanner R. Each point represents the on-spot background of columns 5–10, all of which are below the LOD. Scans were taken in triplicate with a five-minute lag. The scans plotted represent 27 scans/week for five weeks.

**Fig. 6:**
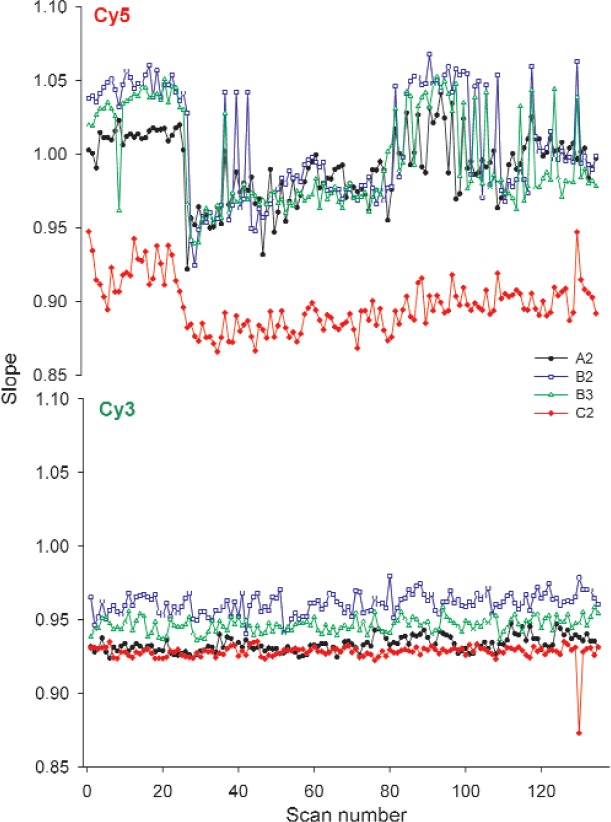
Graphs of the slopes of the heavy use slides over the five weeks of the study taken on Scanner R. Each point represents the slope of a single scan. Scans were taken in triplicate with a five-minute lag. The scans plotted represent 27 scans/week for five weeks.

**Fig. 7:**
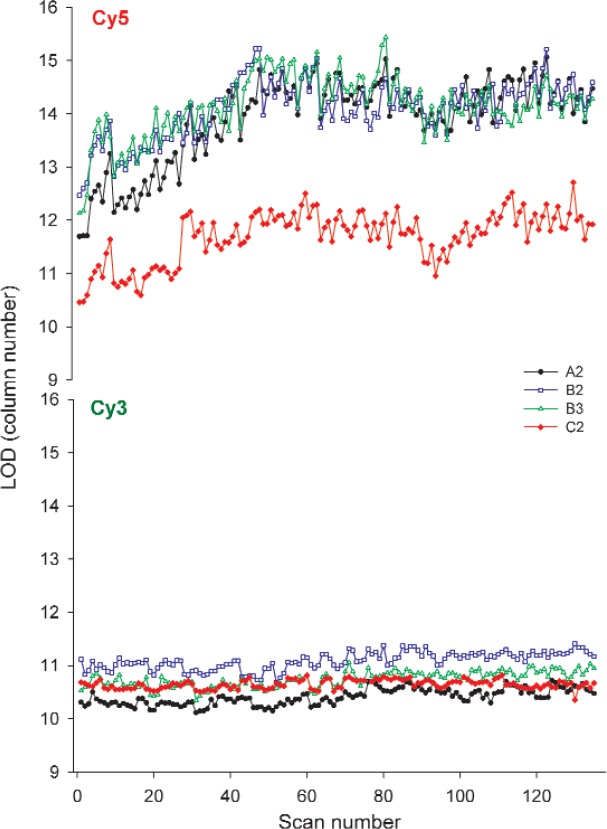
Graphs of the LOD of the heavy use slides over the five weeks of the study taken on Scanner R. Each point represents the LOD of a single scan as a column of dye. The limit of detection was calculated after background subtraction and at a S/N = 3. Scans were taken in triplicate with a five-minute lag. The scans plotted represent 27 scans/week for five weeks.

Each figure of merit was tracked over the five weeks of the study for each of 12 dye slides. The time series charts of the figures of merit for the heavy use slides, A2, B2, B3, and C2, are shown in Figs. 3–7. Slides A2, B2, and C2 were from different lots but were treated the same throughout. Slide B3 was from the same lot as slide B2 but was not stored in a desiccator. Upon examination of the charts, slide-to-slide differences are readily apparent for some of the figures of merit, differences that may be in part attributed to differences in instrument settings. As shown in Table 3, the instrument settings used for the lot C slides varied slightly from those used with lots A and B, in most cases requiring slightly higher gain settings for equivalent signal intensities. The instrument settings used for the two scanners were slightly different based on the optimization performed on the first day of the study but resulted in similar trends over time, as seen on the graphs. (More details are available in Sec. 2.1.)

### 3.2 Figures of Merit

#### 3.2.1 Signal Intensity of a Single Column

**Fig. 8:**
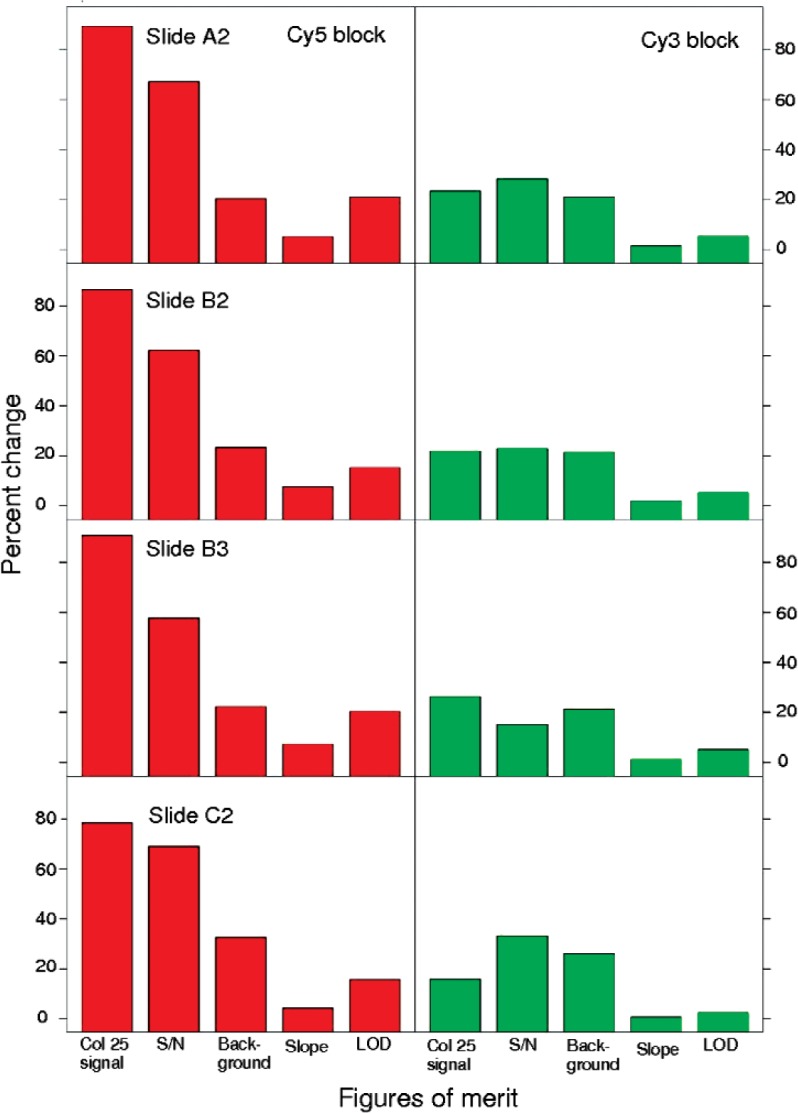
Percent change of the heavy use slides scanned 27 times/week on Scanner R over the five weeks of the study. Percent change was calculated from measurements of the maximum and minimum figures of merit.

Signal intensity of a single column in the linear region was tracked in the red and green dye blocks as the simplest measure of instrument performance over time. Over the five weeks of the study, signal intensity in the Cy3 block of the dye slides was consistent, as shown in Fig. 3. The three scans taken one right after the other are often different from other triplicates, showing changes between morning and afternoon and afternoon and the following day, behavior also observed with the SRM. The percent change of the median signal intensity of the triplicate scans of column 25 in the Cy3 block of the heavy use slides is shown graphically in Fig. 8 and indicates a percent change of between 16–26 % over the course of the study indicative of the stability of the Cy3 dye.

In contrast to the Cy3 block, the percent change in the Cy5 block over the five weeks of the study period for the heavy use slides was between 78–91 %. Considerable scan to scan variability was observed in the Cy5 block despite the five-minute recovery time allowed between each scan. It should be noted that the higher signal observed for the Cy5 block of slide C2 could be attributed to the instrument settings used, which were slightly higher and thus more sensitive than for the lots A and B slides (see Table 3). More importantly for the purposes of this study, the range of values measured and trend of signal intensities over time for slide C2 is closely associated with that of the other heavy use slides. Correlation measures indicate a strong relationship among heavy use slides for both dyes over time. In other words, although the absolute signal intensities of the slides may differ, the heavy use dye slides investigated all exhibit the same pattern of signal intensities over time which indicates the potential of this figure of merit as a measure of scanner instrument performance, in particular for the Cy3 block.

#### 3.2.2 Signal to Noise

Signal to noise (S/N) of column 25 was calculated and tracked for the dye slides over the course of the five-week study. The noise measurement used is a gauge of the variability of the signal among the 12 rows of identical concentration, as well as a measure of how consistently the dye spots are printed from row to row and how well the scanner is able to measure the fluorescence intensity in nominally identical features. If the dye is photostable and printed consistently in the 12 rows changes in the noise measurements should be related solely to instrumental changes.

As seen in Fig. 4, S/N in the Cy3 block was higher than in the Cy5 block, with the measurements of S/N of slide A2 higher than those of the other heavy use slides in the Cy3 block. The increased S/N measurements for slide A2 were due to significantly lower noise relative to the other heavy use slides, and for slide A2 relative to the other lot A slides.

As observed with the other performance characteristics, the data from the Cy5 block was noisier than that of the Cy3 block over the five weeks of the study. Although the S/N percent changes observed for the Cy5 block are less than those for the signal intensity alone, the percent changes are still considerably higher than those in the Cy3 block (see Fig. 8).

Given the consistent S/N measurements observed in the Cy3 block over the course of the study, this figure of merit holds promise for the ability to track scanner performance over time independent of the dye performance. Despite the slide to slide differences, the stability of the S/N of each individual slide in the Cy3 block indicates the potential of this figure of merit. This figure of merit tracks changes in both instrument sensitivity and variability over the 12 features of the chosen column, which is diagnostically useful.

#### 3.2.3 On-Spot Background

The median signal of columns 5–10, the first five columns with the lowest concentrations of dye applied, was examined and considered for inclusion as a figure of merit. The signal in these columns was greater than the background around the spots, commonly known as the local background, but, as seen in Fig. 1, the signal intensities did not increase proportional to the increase in dye concentration. Columns 5–10 are below the limit of detection with the gain and laser power settings used in this study. Based on these characteristics, the signal in these columns was utilized as a measure of on-spot background and tracked for each scan made. An advantage of tracking on-spot background is its independence of the dye concentration; i.e. changes in the dye concentration do not result in increases in the on-spot signal intensity, therefore changes over time in measures of on-spot background should be indicative of scanner performance only.

Having said this, an unexplained phenomenon was evident in columns 5–10 of both the Cy5 and Cy3 blocks for all the slides used in which a gradual decrease in signal from column to column was evident in the columns under surveillance. The change in signal was not as regular as that observed above the LOD in which the signal intensity approximately doubled as the concentration doubled. This signal change can be explained neither by the changes in the dye concentration nor by instrumental changes. For the purposes of this study the signal change in the background region did not appear to affect the data quality.

The independence of the on-spot background signal intensity from the dye performance is evident in the differences between the graphs of the different figures of merit. The graphs tracking background show signs of correlation between the dyes, in contrast to measures within the linear range of the dose-response curve. For example, as seen in Fig. 5, the on-spot background signal intensities of slide C2 in both the Cy5 and Cy3 blocks are highly correlated.

The slide to slide signal intensities also are similar for this figure of merit with close correlation among the median signal intensities of columns 5–10 of the heavy use slides of lots A and B evident in both the Cy5 and Cy3 blocks. Although differing in absolute measures the Cy5 and Cy3 data of the three lots A and B heavy use slides show similar percent changes throughout the five weeks of the study (see Fig. 8). A comparison of Cy3 on-spot background to Cy3 column 25 signal intensity measurements for the heavy use slides shows a notable similarity in which the triplicates of scans can be distinguished based on when the scans were made. As seen with the column 25 signal intensity and with the SRM, measurements of on-spot background in the Cy3 block group together in threes related to when they were measured, although whether this is instrument or material related is unknown.

The graphs of on-spot background of both the Cy5 and Cy3 blocks show promise for use in tracking instrument performance over time. However, because they are a measure of background and below the LOD, they do not give an indication of scanner performance in the range in which most researchers are interested. It is unknown whether changes within the linear region would be detectable when only background is monitored.

#### 3.2.4 Slope

Slope, as a measure of the sensitivity, was calculated from the linear region of the fitted dose-response curve. Since slope is not dependent on the absolute signal intensities, it was anticipated that this figure of merit would be less sensitive to the variability introduced by scanner-related photodegradation, and therefore show potential as a figure of merit. As seen in Fig. 8 in which the percent changes of the heavy use slides on the Scanner R are represented graphically, the slope in both the Cy5 and Cy3 blocks of the dye slides exhibited a smaller percent change over time than any of the other figures of merit. Slide to slide differences were observed throughout the study but the slides followed the same trend over time, as shown in Fig. 6.

Visual inspection of the raw images of the scans (available as tif files in the supplementary material) over time revealed signs of temporary photobleaching in the Cy5 block with fewer columns saturated for the second and third scans of a triplicate. Photobleaching of the dye resulted in a shift in the dynamic range but had a minimal effect on the slope. This relationship was further explored by testing for correlation between the first column used in the linear region, called the start column, and the slope. A small negative correlation was observed, indicating that the greater the start column or higher the lowest concentration in the dynamic range, as in the photobleached scans, the lower the slope. No similar correlations were found in the Cy3 block. An age/lot effect was observed in the lot C slides, the slides printed nine months before use. Much greater variability was observed in the Cy5 start column which varied from column 14 to column 20 while the start column for the lots A and B slides varied between column 17 to column 20. The increased variability in the start column of the lot C slides is another possible indication of the Cy5 dye degradation over time.

The variability in the measurements of the Cy3 slopes for each individual slide over the five weeks of this study is less than 2 % indicating the potential of this figure of merit as a sensitive measure of scanner performance over time. The absolute measurements of the slopes differ from slide to slide but the consistency of the measurements over time bodes well for their use in tracking scanner performance if a single slide is consistently used with one instrument.

#### 3.2.5 Limit of Detection (LOD)

The LOD, calculated as the column with the minimum concentration detectable with 99 % certainty, was monitored as a possible figure of merit. This figure is calculated from the fitted dose-response curve (background-corrected signal versus column number) as the column where the signal is equal to three times the noise. Changes in the scanner that affect the slope, background, intercept, and noise (standard deviation of the background) will manifest as a change in the LOD, making it a potentially useful overall performance measure.

As shown in Fig. 7, the LOD in the Cy5 block of the lots A and B heavy use slides increased gradually over the first two weeks of the study before leveling off, matching corresponding signal changes in the column 25 signal intensity. For the heavy use slides under the conditions used in this study, the observed LOD of the Cy3 block was consistent and stable throughout the observed time period, with a percent change of 5 % or less. For both blocks, a strong negative correlation between the LOD and intercept was evident suggesting that changes in the LOD could be attributed to a measure of apparent sensitivity as the dynamic range shifted. This was consistent with the observation that the LOD of the Cy5 block often shows a systematic increase in the triplicate measurements.

Based on the stability of the LOD in the Cy3 block over the five weeks of the study this figure of merit may be worthy of use for tracking microarray scanner performance. The LOD as a multi-component measurement of scanner performance makes it attractive as a figure of merit.

### 3.3 Factors of Significance

**Fig. 9:**
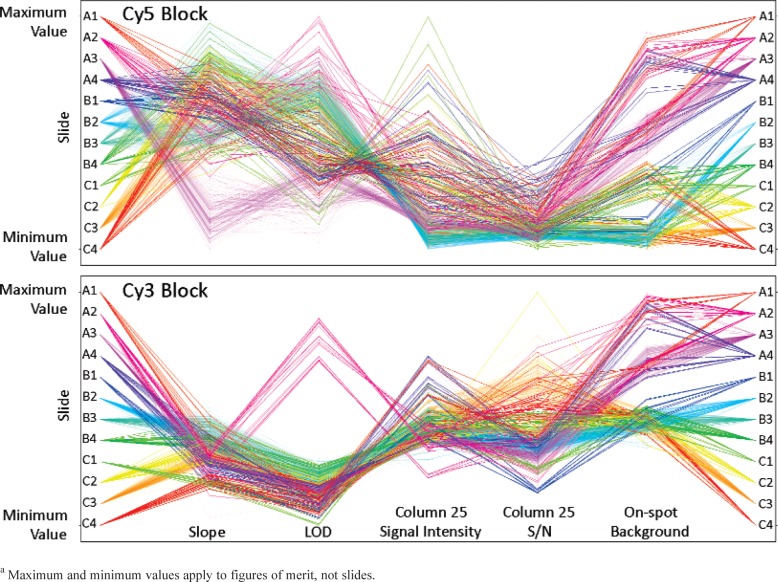
Figures of merit of all 12 slides for all scans taken on Scanner R during the five week study. Each slide is represented by a different color with a single line representing each scan, thus the heavy use slides have 135 lines, the standard use slides have 15 lines and the minimal use slides have 6 lines. The lines represent each scan and are used to visually indicate each data point for each figure of merit. Each figure of merit is normalized so the range shown is the range of values exhibited by the figures of merit in the study for both Cy5 and Cy3 dye blocks.

A number of factors and their influence on the figures of merit were investigated, as shown in Table 2. As is evident in Fig. 9 in which all the figures of merit are graphed for scans of all the slides on Scanner R, slide to slide differences are obvious, regardless of the factor under study. Analysis of variance (ANOVA) of all slides confirmed that the slides were statistically different from each other with p-values lower than 0.05 no matter the treatment. For example, when the figures of merit of desiccated versus room air slides were compared, the slides were different, as they were when standard use slides (n = 15) were compared to heavy use slides (n = 135). However, since a laboratory would typically use a single slide for performance qualification the within-slide trends over time are of greater interest and importance than the slide to slide differences. Observations of the same trends for most of the slides supported the experimental design, however, without experiments comparing slides treated exactly the same the effects of different factors on slide behavior cannot be separated from the slide to slide differences.

While acknowledging that slide to slide differences were not compared it is still possible to investigate the effects of the factors. The number of scans made over the course of the five weeks of the study was investigated by scanning some slides daily (n = 135), some weekly (n = 15), and others only at the beginning and end of the study (n = 6) on Scanner R. To determine the effect of daily scans versus weekly scans versus monthly scans on the figures of merit the percent change between the median values of the triplicate of scans made in quick succession taken on Day 1 of the study and the median of the last scan triplicate of the study were compared. If the number of scans made adversely affected the dye stability this would be evident in an increased start-to-finish percent change for the heavy use slides. Analysis of the start-to-finish percent change revealed no significant difference in the green block for the heavy use slides compared to the standard use slides and the minimum use slides and a slight increase in percent change for the heavy use slides in the red block. The number of scans performed does not appear to have a deleterious effect on the figures of merit for Cy3 tracked in this study.

Recovery time, or the amount of time between scans, had little impact on the figures of merit. Although some triplicate measurements exhibited a cluster pattern in which the signal was similar for the three scans in a row, no pattern emerged when increased recovery time was allowed. When the percent change among triplicates is compared to the percent change between the first scan of the morning and first of the afternoon, or to scans taken on Friday to scans taken on Monday the differences are not significant, although changes in the Cy5 block are almost always greater than in the Cy3 block.

The effect of the age of the slide was investigated through the use of slides printed approximately nine months before the study, the lot C slides, compared to slides printed approximately one month before the study, lots A and B. However, it was not possible to determine if the observed differences were due to differences between lots or age. Given this caveat, as shown on the graphs of the figures of merit, Fig. 3–7, differences between the older and newer slides are evident. Some of the differences, for example the increased background intensity of the lot C slides, can be attributed to instrument settings (See Sec. 2.3). In the Cy3 block for most figures of merit the lot C slides appear similar in behavior to the newer slides whereas in the Cy5 block the lot C slides are usually visually separated from the newer slides, perhaps indicative of the Cy5 dye degradation over time. More importantly, although the absolute values of the lot C slides were often different than those of the lots A and B slides in the Cy5 block, the behavior of the figures of merit for all the slides no matter the age was similar. In other words, although the starting point of the older slides differed from those of the newer slides the signal stability was comparable over the five weeks of the study.

Another factor investigated in this study was the effect of storing the slides under desiccation versus storing the slides under ambient conditions in which the humidity varied between 21 % to 38 %. Although statistical analysis as well as visual inspection of the graphs of the slides showed differences between the desiccated and non-desiccated slides the differences cannot be attributed solely to the storage conditions. The slides stored in room air did not exhibit more variability or greater dye degradation over the course of the study leading to the conclusion that lack of desiccation did not have a discernable effect on the figures of merit investigated in this study. It should be noted that this study took place in March and April, a time of the year that is typically cool and dry in the location of the study. It is possible that the increased humidity experienced in the summer months may contribute adversely to the signal stability.

**Fig. 10:**
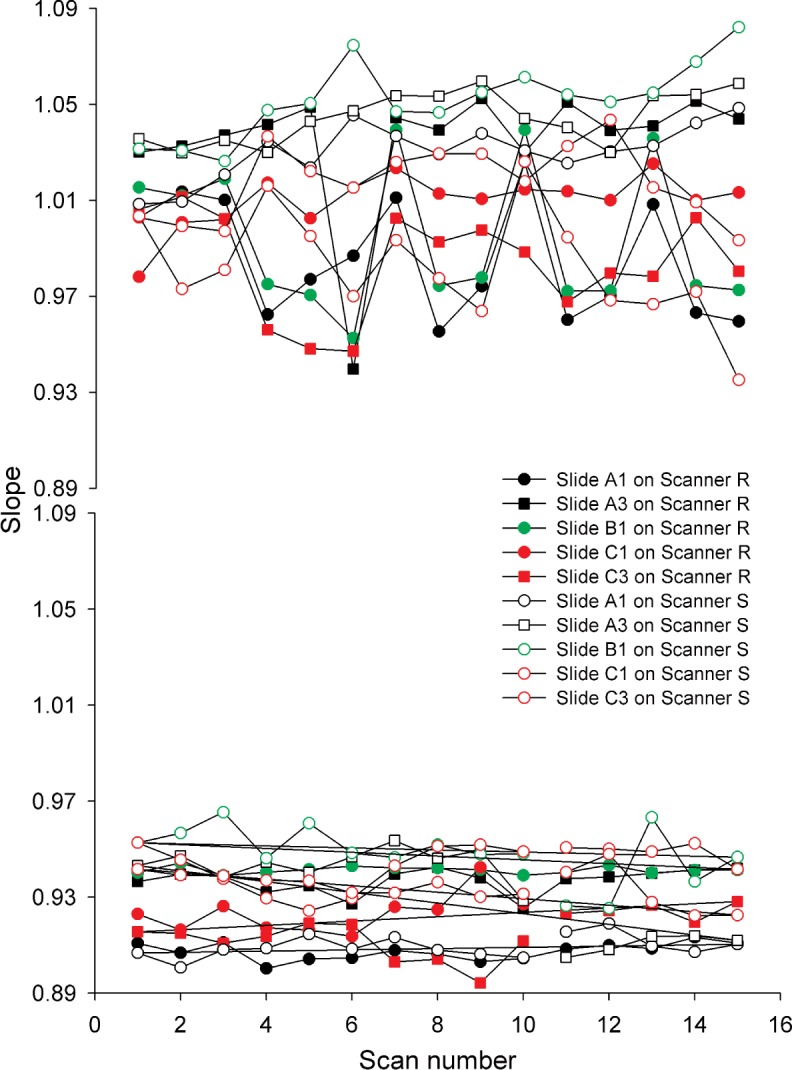
Graphs of the slopes of the standard use slides scanned on Scanner R (filled-in symbols) and Scanner S (open symbols). Scans were made in triplicate with a five-minute lag once a week for five weeks. The scans plotted represent 3 scans/week for five weeks.

One of the ultimate goals of this study is to provide methods for comparison of data from different scanners. A graph of the slopes of the five standard use slides which were scanned equal times on both scanners and on the same days is shown in Fig. 10. Slope measurements from the two scanners are not easily distinguishable and the behavior of the slides over the five weeks of the study on the two scanners appears similar. To compare how much the slope changed over the time period of the study, the percent change was calculated using the maximum and minimum measurements of the triplicate medians made over the five weeks of the study. Percent changes between the two scanners for the slope, LOD, and column 25 signal intensity of the standard use slides were similar for the two instruments. However the percent changes of the S/N of the Cy3 block and on-spot background for both blocks were consistently greater for all the standard use slides when scanned on Scanner S. Since the background is the only figure of merit unaffected by the Cy5 dye performance, it seems likely that if the dye had been more stable the S/N of the Cy5 block would have also shown a greater percent change on Scanner S. The greater variability observed with Scanner S for these two figures of merit can be attributed to instrumental differences. Such differences are important to be aware of, and highlight the importance of using the same scanner throughout a study, but do not necessarily affect instrumental stability in experimental scanning.

### 3.4 Scanning and Slide Anomalies

No known instrumental problems occurred during the five weeks of the study, and only one scanning anomaly was observed. On the last day of the study, Day 30, slide C2 was the second slide of the four heavy use slides to be scanned. As observed in the graphs of signal intensity, S/N, on-spot background, and slope, the first scan of slide C2 that morning, scan number 125, resulted in incongruities in the Cy3 figures of merit including a lower than expected signal intensity, S/N, on-spot background, and slope. An investigation of the noise associated with these measurements indicated much greater variability among the 12 rows than usual. Because the figures of merit for the following two scans of the morning triplicate of slide C2 were within the normal range, one can only call this initial measurement aberrant, with cause unknown but perhaps related to the age of the slide. Changes in the instrument were probably not the cause since scans of the other slides were unremarkable as were the second and third scan of slide C2. If the additional scans or scans of other slides had also indicated irregularities, this would have been cause for further investigation. As the issue appeared to resolve itself after this one scan no further action was taken or seen as necessary.

This anomaly was detected in the signal intensity, S/N, on-spot background, and slope graphs of the Cy3 block, but only on the Cy5 block on-spot background graph. Since the Cy5 on-spot background is the single figure of merit that is independent of the dye it is likely that the aberration observed within the Cy3 data also occurred in the Cy5 data but was not detected due to the inherent noise of the fluorescence measured from the Cy5 dye; i.e. the aberration in the Cy5 data was not great enough to be differentiated from the usual Cy5 variability. This incongruity illustrates how each of the figures of merit investigated has the potential to provide insight into instrument performance as well as the effect of the instability of the Cy5 dye.

Of the 12 slides used in the study Slide C3, a standard use slide from the lot printed nine months prior to use, was distinguished from the other slides due to its high background noise. On Scanner R, a ten-fold increase in the standard deviation of the on-spot background of the Cy5 and Cy3 blocks of slide C3 was observed between the period of the first three weeks of the study to a second period of weeks four and five. On Scanner S the standard deviation of the on-spot background of slide C3 was much higher than that of the other lot C slides throughout the five weeks of the study. This deviation from the other slides in the same lot was not immediately evident because the high background noise level only affected the LOD. None of the other parameters tracked including the variability of the figures of merit varied significantly among the slides except for the low column 25 signal intensity noise of slide A2 discussed earlier. The source of the increase in the background of slide C3 was not determined, but appears to be slide-related rather than instrument-related.

### 3.5 Study Implications

The purpose of this study was to investigate microarray scanner performance over time and to assess the dye slides as potential tools for instrument performance qualification. Since it is realistic to assume that only one dye slide would be used in a laboratory at a time and a single scanner used throughout a study, the performance of a single dye slide over time is more critical than slide to slide reproducibility or scanner differences. As demonstrated by the graphs, the performance of the figures of merit in the Cy3 dye block over the five weeks of the study is consistent on both scanners, and appears to be useful for tracking scanner signal stability and performance. The implications of tracking figures of merit for Cy3 and using them to assess scanner performance at wavelengths used with Cy5 cannot be assessed from these data.

In a practical lab setting for microarray scanner performance qualification, the user would establish the behavior of the slide and scanner by obtaining baseline data. This baseline study of perhaps a week with daily scans would also ascertain that the behavior on different scanners may be distinct but that each is equally valid. After collecting baseline data, scanner performance could be monitored on a weekly basis, or as needed, bracketing experimental scans to assure microarray scanner performance in the Cy3 channel and presumably the Cy5 channel. The techniques laid out herein provide a method for verifying that the scanner performance is similar to when it was previously measured. Although not yet able to provide quantitative uncertainty measures of the variability the microarray scanner is contributing to an experiment, this initial study provides information that could result in performance qualification of a microarray scanner with a commercially available tool.

## 4. Conclusion

Qualification of microarray scanner performance entails providing evidence that the scanner is performing the same on one day as it did on the day of previous use using predetermined specifications and quality attributes. Such instrument qualification will require baseline information as a history of how the instrument has acted in the past under normal working conditions so comparisons can be made and documented. Through the use of a Cy5/Cy3 dye slide with successive dilutions, figures of merit including signal intensity, S/N, on-spot background, slope, and LOD can be tracked as an ongoing check of scanner behavior and signal stability. Use of a single dye slide with a single microarray scanner in the manner described in this study can provide information qualifying scanner performance and monitoring the scanner as a source of experimental variability.

## Figures and Tables

**Table 1:** Figures of merit used for tracking microarray scanner performance in this study

	Definition
Column 25 signal intensity^a^	Mean signal intensity of 12 spots at a given concentration
Column 25 signal to noise (S/N)	Mean signal intensity of column 25 relative to the standard deviation of the 12 spots of column 25
On-spot background	Median signal of spots in columns 5–10, all of which are below the LOD
Slope (sensitivity)	Signal intensity relative to concentration (measured as column number)
Limit of detection (LOD)	Measure of the smallest amount of dye (column number) that can be detected at given conditions

a Column 25 was chosen due to its position in the middle of the linear region.

**Table 2:** Parameters investigated to understand their effect on the figures of merit under consideration for use for microarray scanner performance qualification

Factors investigated for their effects on the figures of merit	Investigation parameters
Number of scans/slide	• heavy use scanned 6x/day: Slides A2, B2, B3, C2
	• standard use scanned 6x/week: Slides A1, A3, B1, C1, C3
	• minimal use scanned 6x/study: Slides A4, B4, C4
Recovery time	• five minutes
	• several hours to two days
Time between slide production and use	• Lots A and B: slides used within three months of printing; within warranty period
	• Lot C: slides printed nine months before use; warranty expired
Slide storage conditions	• in desiccator: Slides A1, A2, A4, B1, B2, B4, C1, C2, C4
	• under ambient conditions: Slides A3, B3, B4, C3
Scanners	• Scanner R used five days/week
	• Scanner S used one day/week

**Table 3:** Instrument settings used for dye slides^a^

		Lots A and B		Lot C (printed approximately nine months before use)	
		Cy5	Cy3	Cy5	Cy3
Scanner R	Gain (V)	725	500	800	540
	Laser power (%)	33	33	33	33
Scanner S	Gain (V)	550	500	600	500
	Laser power (%)	20	5	20	5

a The instrument settings were chosen based on saturation of 4–6 columns, as recommended by the dye slide manufacturer. Settings were kept constant throughout the 5-week study.

## Footnotes

1 Certain commercial equipment, instruments, or materials are identified in this paper to foster understanding. Such identification does not imply recommendation or endorsement by the National Institute of Standards and Technology, nor does it imply that the materials or equipment identified are necessarily the best available for the purpose.
